# Disrupted chromatin architecture in olfactory sensory neurons: looking for the link from COVID-19 infection to anosmia

**DOI:** 10.1038/s41598-023-32896-8

**Published:** 2023-04-11

**Authors:** Zhen Wah Tan, Ping Jing Toong, Enrico Guarnera, Igor N. Berezovsky

**Affiliations:** 1 Agency for Science, Technology and Research (A*STAR), Bioinformatics Institute (BII), 30 Biopolis Street, Matrix, Singapore, 138671 Republic of Singapore; 2 Department of Biological Sciences (DBS), National University of Singapore (NUS), 8 Medical Drive, Singapore, 117597 Singapore; 3 Present Address: Computational Drug Discovery, EMD Serono Research and Development Institute, Merck KGaA, 45A Middlesex Tpke, Billerica, MA 01821 USA

**Keywords:** Biophysics, Computational biology and bioinformatics

## Abstract

We tackle here genomic mechanisms of a rapid onset and recovery from anosmia—a potential diagnostic indicator for early-stage COVID-19 infection. Based on previous observations on how olfactory receptor (OR) gene expression is regulated via chromatin structure in mice, we hypothesized that the disruption of the OR gene expression and, respectively, deficiency of the OR function can be caused by chromatin reorganization taking place upon SARS-CoV-2 infection. We obtained chromatin ensemble reconstructions from COVID-19 patients and control samples using our original computational framework for the whole-genome 3D chromatin ensemble reconstruction. Specifically, we used megabase-scale structural units and effective interactions between them obtained in the Markov State modelling of the Hi-C contact network as an unput in the stochastic embedding procedure of the whole-genome 3D chromatin ensemble reconstruction. We have also developed here a new procedure for analyzing fine structural hierarchy with (sub)TAD-size units in local chromatin regions, which we apply here to parts of chromosomes containing OR genes and corresponding regulatory elements. We observed structural modifications in COVID-19 patients on different levels of chromatin organization, from the alteration of whole genome structure and chromosomal intermingling to reorganization of contacts between chromatin loops at the level of topologically associating domains. While complementary data on known regulatory elements point to potential pathology-associated changes within the overall picture of chromatin alterations, further investigation using additional epigenetic factors mapped on 3D reconstructions with improved resolution will be required for better understanding of anosmia caused by SARS-CoV-2 infection.

## Introduction

The COVID-19 pandemic, caused by the SARS-CoV-2 coronavirus, has placed significant strain on medical resources worldwide, and early detection and isolation has been the primary strategy by which, research establishments and governments worked to contain the disease spread. To enable early detection, surveys performed on early symptoms of SARS-CoV-2 infection uncovered anosmia as a useful predictor of COVID-19 infection^1,2^, showing higher positive and negative predictive values than other flu-like symptoms^3,4^. However, the mechanisms behind the rapid onset of anosmia remained controversial: as a large proportion of COVID-19 patients with anosmia did not experience nasal congestion^5,6^, the reasons behind impaired olfaction apparently extend beyond physical obstruction of odorants in the olfactory epithelium (OE). The SARS-CoV-2 virus enters human host cells via a mechanism similar to the SARS-CoV virus: the proteolytic cleavage of the viral spike protein by TMPRSS2 and entry initiation by binding to the host ACE2 receptor^7^. Since olfactory sensory neurons (OSNs) do not express ACE2 and TMPRSS2 genes at significant levels, it has been proposed that viral infection of non-neuronal cell types in the olfactory epithelium (OE) are the main factors behind impaired olfaction^8^. Among these, the sustentacular (SUS) cells that provide structural support in the OE expression of high levels of both viral entry and receptor genes^8,9^, and infection of SUS cells may impair metabolic and structural functions that are critical for proper OSN functioning^10,11^. Noteworthy, these changes are not likely so drastic as to lead to OSN cell death, as most anosmia patients recover their sense of smell within 1–2 weeks^12–15^, shorter than the time required for OSN replacement and maturation, and axon/cilia growth^16–18^. On the other hand, inflammatory response of the innate immune system in the OE may also contribute to impaired olfaction, as several studies detected high levels of the pro-inflammatory cytokines TNF-α and IL-6 in the OE of SARS-CoV-2 infected patients^19,20^.

While many studies linked released cytokines to changes in the cellular microenvironment that can interfere with OSN function or induce premature apoptosis^19,21^, a few have focused on how inflammatory response in the OE may affect olfactory function at the level of gene expression. For example, in a mouse study, sterile induction of innate immune signaling in the OE was associated with diminished odor discrimination^22^. The OSNs showed significantly reduced expression of olfactory receptors genes (ORs), which encode G protein-coupled receptors that activate olfactory signal transduction pathways on odorant ligand binding. This suggests that olfactory dysfunction in response to inflammation may be traced to altered OR gene expression regulation in OSNs. The human OR gene repertoire consists of 376 functional genes distributed across 18 chromosomes, and it can be categorized into distinct phylogenetic groups, Class I and Class II, by sequence homology and functional specificity - the former is presumed to detect hydrophilic odorants and the latter is activated by hydrophobic ones. Most OR genes are located in clusters up to ~ 1 Mbp in size. It has been widely accepted that in the maturation process of OSNs, the neurons begin to exhibit specialized OR gene expression, and each mature OSN (mOSN) expresses only one allele of one randomly selected OR gene^23,24^. With gene translocation ruled out as a possible mechanism for OR selection^25^, current models based on mouse studies propose that the control of mOSN OR gene expression is achieved via multiple levels of chromatin organization. At the whole-genome level, immuno-fluorescence studies have found that olfactory sensory neurons exhibit an inverted nuclear architecture. While typical cells are organized with constitutive heterochromatin (cHC) localized on the nuclear lamina in the nuclear periphery, OSN genes show an aggregation of a large cHC block away from the lamina, surrounded by facultative heterochromatin (fHC)^26^. The observed tendency for OR genes to colocalize in the cHC or fHC regions may facilitate the selection of a single allele for expression^27,28^. This inverted architecture appears to also be mediated by the downregulation of lamin-B receptors (LBR) in OSNs, as restoring LBR gene expression led to a decondensation of the cHC block and a concomitant disruption of OR gene aggregation and expression^27^. At a finer structural scale, epigenomics and reporter assays have also identified a set of enhancers located in the vicinity of mouse OR genes (termed the Greek Islands)^29^. These regulatory elements were observed to physically interact with activated genes and with one another, even across different chromosomes^30,31^. In view of these two levels of chromatin organization, a plausible picture of OR gene regulation in OSNs may consist of the aggregation of OR genes in the HC block, potentially enabling the selection of one OR gene allele to be unfolded and activated through a looping interaction with an interchromosomal enhancer hub^31^.

While non-cell-autonomous disruption of interchromosomal OR compartments leading to their downregulation was recently shown in human^20^, neither direct link to COVID-19 caused anosmia, nor details of structural changes in chromatin were yet obtained. We tackle here a question about structural foundation of the OR genes regulation, seeking for the structural link from COVID-19 infection to anosmia, which would be complementary to results of the above work. To understand how SARS-CoV-2 infection may lead to anosmia through chromatin structure disruption and dysregulation of OR gene expression, we began by using publicly available Hi-C data of OSNs of SARS-CoV-2-infected and control human subjects^20^, obtaining the whole-genome reconstructions of the chromatin ensemble in each subject^32^. Focusing on the detailed organization of OR genomic clusters, we then analyzed Hi-C data to identify fine structural units of chromatin and potential functional links between them. We adopted a two-pronged approach in characterizing chromatin structural differences in SARS-CoV-2-infected patients and controls, on one hand studying large-scale structural shifts by spatial reconstructions at the genome level, and on the other hand zooming in to fine-scale organization of key gene clusters. Firstly, we use a Markov state model to identify coarse Mb-scale structural units^33^, then apply a stochastic embedding procedure^32^ to obtain genome-level reconstructions of the chromatin ensemble. Secondly, to understand chromatin organization and packing at the local level we devised a novel method for identifying localized structural units at the ~ 30 to 200–500 kbp scale, mapping out how strong links between these units shape physical packing of the chromatin fibre from chromatin loops^34–36^ to (sub)TADs^37^. Olfactory receptor gene regulation is recognized as involving inter-chromosomal processes in mice^31^, with olfactory receptor genes organized in multiple clusters spread across multiple chromosomes in both mice and humans. This work provides a first look at how normal human OSNs exhibit similar features of chromatin organization to that observed in studies of normal mouse OSNs, highlighting how the disruption of OSN chromatin structure may be an important structural basis of the development of anosmia in SARS-CoV-2 patients.

## Methods

### Reconstruction of 3D chromatin ensemble structure

**Figure 1:**
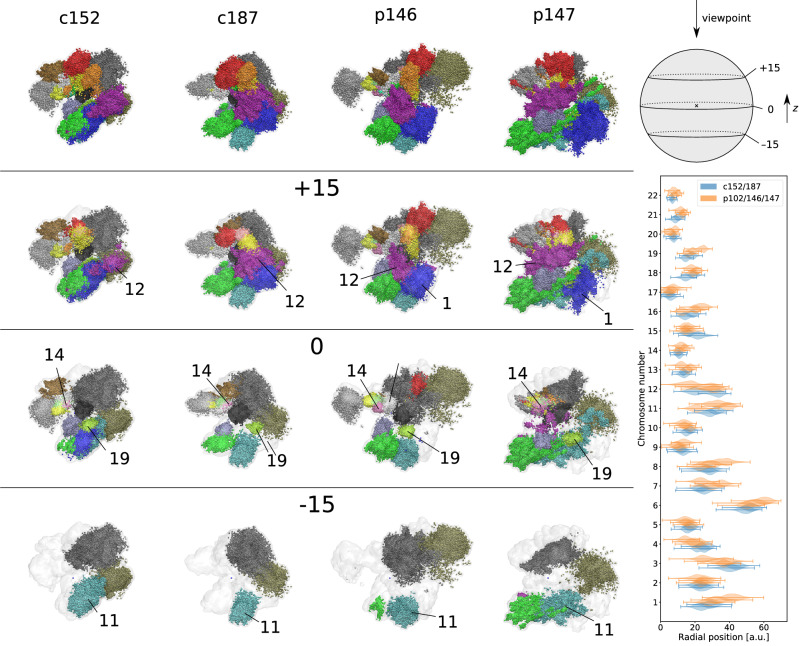
Reconstructions of whole-genome chromatin ensemble for olfactory sensory neurons in SARS-CoV-2-infected patients and controls. Left, top row: Full reconstructions colored by chromosomes are shown for controls (c152, c187) and patients (p102, p146, p147). (Bottom left) Subsequent rows below are cross-sectional views of the respective reconstructions, at the positions indicated by the schematic (top right), where $z=0$ intersects the centroid. The approximate centers of chromosomal territories 1, 9, 11, 12, 14, and 19 are indicated on the cross sections. Radial distributions of chromosomal territories, comparing the same chromosomes on control (blue) and patient (orange) samples.

We investigate here large-scale changes in the whole-genome chromatin captured in Hi-C chromatin interaction data for OSNs from 4 SARS-CoV-2-infected patients and 2 control subjects, by reconstructing of the chromatin ensemble structure (Fig. 1 and Supplementary Figure S1). This approach consists of two steps, by first (a) identifying megabase-level structural partitions using a Markov State Model (MSM) of Hi-C interactions^33^, and second (ii) obtaining ensemble reconstructions at the level of these partitions using a stochastic embedding procedure (SEP)^32^, as implemented in software pipelines published previously.

In the first step, we partition the chromatin interaction network using a MSM approach. By modelling the diffusion of small molecules in densely packed chromatin, the MSM enables one to partition the genome into structural units, which provides a suitable level of coarse-graining of genome structure as captured by the Hi-C experiment. For each sample, we identified more than 800 structural units with average size 3Mbp across the genome. Then, to obtain spatial embeddings of chromatin ensemble structure, we define a matrix of effective interactions between the structural partitions. These effective interactions are subject to stochastic fluctuations in the cell population, and we applied the SEP method^32^ to reconstruct the chromatin ensemble through sampling of structural variations between and within individual structural units. Briefly, we identified structural units in the genome by applying a MSM partitioning procedure^33^ to Hi-C data using a metastability criterion of $\rho <0.9$, yielding 950, 901, 850, 952, 800, and 903 structural units for samples c152, c187, p102, p116, p146, and p147, respectively. Effective interactions between the structural units are defined by coarse-graining the Hi-C matrices. To map effective interaction matrices to ensemble structures, we applied the Stochastic Embedding Procedure we developed previously^32^, which uses a distance geometry approach for spatial embedding of effective interaction data subject to stochastic fluctuations. We obtained $N=100$ samples of fluctuations in effective interactions using a gaussian model with a strength factor $\alpha =0.2$. The sampled interaction matrices were normalized using the SCPNs algorithm described previously^32^, before using spatial embedding to obtain positions of individual structural units. We then modelled each structural partition as a spherical crumpled globule with radius $r$ scaling with partition size $s$, $r\sim {s}^{0.6}$, representing intra-partition structural variability by random sampling of points within the partitions at 1Mbp intervals. The merged samples represent regions of space accessible to individual partitions in the ensemble of chromatin structural states. Without information on the gender of subjects, we have focused structural analysis only on somatic chromosomes 1–22 in this work.

### Robustness of chromatin structural variation between samples

Considering limited number of samples available for the analysis, we resorted to evaluation of the robustness between them. In future work, while it is desirable to have more samples, robustness should be still evaluated to select out obvious outliers. To evaluate the robustness of ensemble reconstructions and the significance of changes in chromatin structure, we repeated the 2-steps reconstruction procedure for each Hi-C sample by performing 3 independent MSM partitioning runs (MSM replicates), followed by 2 independent SEP ensemble samplings for each obtained partitioning (SEP replicates). The 6 replicate reconstructions per Hi-C sample are shown in Supplementary Figure S2 along with the network of chromosomal intermingling, and we show a quantitative comparison of these ensemble reconstructions in Supplementary Figure S3. The bottom-left of the latter shows differences between reconstructions of the same Hi-C sample reflected in shifts in the distance correlation functions g(r), while the top-right shows the large-scale changes in chromosomal mixing fractions between all reconstructions.

Firstly, as distance correlations $g(r)$ capture more detailed information on chromosome morphology, we used the histogram difference $\Delta g(r)$ (see “Materials and methods” section) to quantify random structural shifts between reconstructions of the same Hi-C sample (see bottom left of Supplementary Figure S3). We found essentially no structural difference between SEP replicates, indicating that the sampling of interaction fluctuations is sufficient to yield robust ensemble representations, which point to potential chromatin conformational changes related to the regulation of genome expression. Between MSM replicates of the same Hi-C datasets (except for p146), we observed only weak differences in $g(r)$ owing to minor shifts in chromosomal positioning. For p146, visual inspection of the structures and g(r) correlation functions of MSM replicates showed that the reconstructions maintained consistent relative positioning of chromosomal territories, despite slightly larger variation in bulk distances between non-intermingling chromosomes that led to the larger histogram differences $\Delta g(r)$ seen in Supplementary Figure S3. Secondly, to study large-scale structural differences between samples, we focused on changes in chromosomal intermingling and quantified the mixing fraction ${m}_{ij}$ of chromosome $i$ in chromosome $j$ as the fraction of chromosome $i$ located within a cutoff distance 1 a.u. from chromosome $j$ (see “Materials and methods” section). Comparing all 231 chromosome pairs across all 36 ensemble reconstructions, 69 chromosomal pairs consistently showed no observable intermingling. Among the remaining 162 chromosomal pairs, Supplementary Figure S3 shows that mixing fractions were largely consistent between MSM and SEP replicates of each Hi-C sample, compared to the differences between Hi-C samples. While some differences exist between the 2 control samples, we observed a higher degree of heterogeneity between the 4 patient samples (Kruskal–Wallis H-test on the first principal components of control and patient samples, yielding *p* values 4 × 10^−3^ and 2 × 10^−4^), with p147 and p116 showing greater and less chromosomal intermingling respectively. Importantly, Supplementary Figure S3 shows significant differences between controls and patients in chromosomal intermingling, with several chromosome pairs showing strong shifts between control and patient chromatin states (38 chromosomal pairs with Mann–Whitney U-test *p* value < 0.1%). The ensemble reconstruction procedure implemented was thus able to capture structural variation within the control and patient sample groups, and to identify consistent structural changes in OSNs for SARS-CoV-2-infected patients.

### Quantifying intermingling between chromosomal territories

We quantified the degree to which chromosomal territories intermingle using ensemble reconstructions obtained from the stochastic embedding procedure. To that end, we defined the mixing fraction ${m}_{ij}$ from chromosome $i$ to chromosome $j$ as the fraction of sampled points in $i$ within a cutoff distance of 1.0 a.u. (corresponding to a physical distance of approximately 350 nm) from sampled points in $j$. In this work, each chromosome is represented by 5000 to 25,000 points (proportional to chromosome size), and a mixing fraction of ${m}_{ij}\ge 10\%$ constitutes significant intermingling. For two ensemble reconstructions ${r}_{1}$ and ${r}_{2}$, we quantified the difference between mixing fractions by the simple difference $\Delta {m}_{ij}^{{r}_{1},{r}_{2}}={m}_{ij}^{{r}_{1}}-{m}_{ij}^{{r}_{2}}$. Positive delta $\Delta {m}_{ij}^{{r}_{1},{r}_{2}}$ indicates that the chromosomes *i* and *j* show more intermingling in *r*_*1*_ than in *r2*, while negative values indicate the reverse trend. The upper half of Supplementary Figure 3 indicates the difference delta m between mixing fractions for any two reconstructions indicated by the row and column. As an example, the negative values (blue) in comparisons for all other samples with p147 indicate that chromosomal intermingling was generally reduced in p147.

### Distance correlation functions g(r) of single chromosomes or chromosome pairs

**Figure 2:**
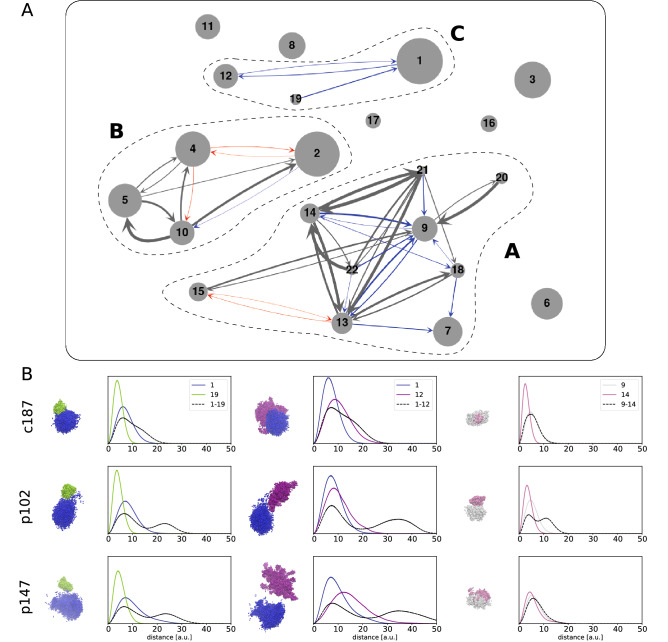
Differences in chromosomal intermingling between control and patient samples. (**A**) Chromosomal intermingling network in the ensemble reconstructions. Chromosomes are represented as nodes with radius scaling with chromosome size. Edges connect chromosomal pairs that intermingle strongly in all controls only (blue), all patients only (red), or in all controls and patients (grey); edge width represents the average mixing fraction observed in corresponding samples. Three chromosomal groups can be identified in the network, as marked by dashed lines. (**B**) Typical chromosomal morphology and distance correlation functions $g(r)$ of chromosome pairs 1–19, 1–12, and 9–14 in controls and patients. Chromosomes adopt the same color convention as in Fig. 1: 1 – blue, 9 – white, 12 – purple, 14 – pink, 19–lime green.

To capture more detailed information on chromosomal morphology, we determined the pairwise distance correlation functions $g(r)$ for chromosome pairs, as well as for individual chromosomes (Fig. 2 and Supplementary Figure S4). The distance correlation ${g}_{ij}\left(r\right)$ for chromosomes $i$ and $j$ is defined as the probability density that two randomly sampled points in the chromosome pair is separated by a distance $r$: from ensemble reconstructions, we computed the $g(r)$ functions as a normalized histogram of sampled pairwise distances. The single-chromosome correlation functions ${g}_{i}\left(r\right)$ are defined and computed similarly, considering only sampled points in one chromosome. The difference between distance correlations in two ensemble reconstructions ${r}_{1}$ and ${r}_{2}$ is quantified by the histogram distance:

$${\Delta }g_{ij}^{{r_{1} ,r_{2} }} \left( r \right) = \int {\left| {g_{ij}^{{r_{1} }} \left( r \right) - g_{ij}^{{r_{2} }} \left( r \right)} \right|} dr$$

### Human olfactory receptors, OSN markers, and putative enhancers mapped from mouse data

We retrieved a list of identified human olfactory receptor (OR) genes from the HORDE database^38^, which contained a total of 376 functioning genes, after removing 439 pseudogenes. Genomic coordinates for each gene were then identified on the hg38 human reference genome. The OR class is identified by the gene family ID: ID numbers 51 to 56 are categorized as Class I, while all others belong to Class II. A list of 15 marker genes for mature human OSNs was retrieved from (24). A list of 35 mouse olfactory gene enhancers, termed Greek Islands, was identified by the Lomvardas Lab (29). We mapped the genomic coordinates of these enhancers to the human reference genome by successive application of the UCSC LiftOver tool (https://genome.ucsc.edu/cgi-bin/hgLiftOver), from 9 to 10 mm, then from 10 mm to 38 hg. Among the 25 successfully mapped regions, we further required that putative enhancers (PEs) be located at most 1 Mbp from the nearest human OR genes, yielding a final list of 15 PEs. The full list of mice enhancers’ mapped genomic coordinates, and proximity to OR genes are provided in Supplementary Table S1.

### Local structural unit analysis of fine chromatin structure

**Figure 3:**
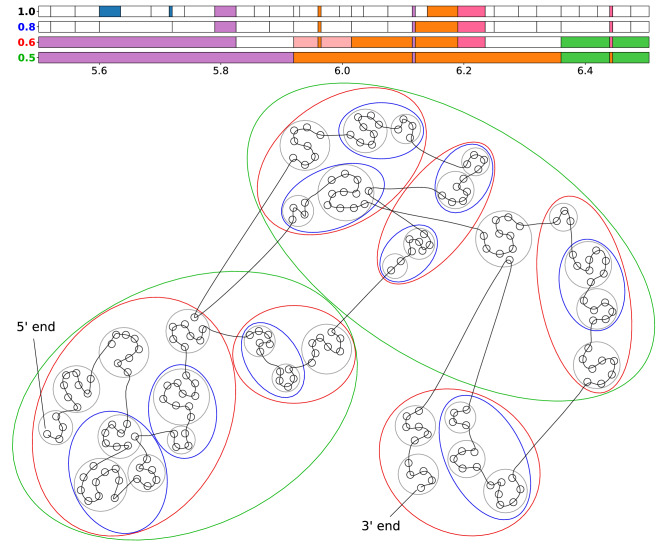
Iterative procedure for identifying fine structural hierarchy in chromosomes. This figure shows structural units identified at various levels of hierarchy in a 1 Mb region (5.5–6.5 Mb) of chromosome 11. In the network representation, individual 5 kb bins/loci in this region are represented by black circular nodes, with a chromosomal trace overlaid to show the relative genomic locations of each locus. Beginning by considering each locus as an individual structural unit, we iteratively join pairs of structural units with the highest interacting energy ratios $r$, to a point where the largest energy ratio ${r}_{max}$ falls below 1.0, yielding structural units denoted by the grey circles. Continuing with this procedure, we obtain different levels of hierarchy when ${r}_{max}$ falls below 0.8, 0.6, and 0.5, which give structural units represented by the blue, red, and green ellipses, respectively. 1D representations of the structural units (in the top) obtained at each level of hierarchy are denoted by the value of ${r}_{max}$ on the left. Units composed of a single contiguous genomic segment are shown as white horizontal bars in the plot, while units composed of non-contiguous segments are color-coded accordingly: e.g., the two blue segments and the two orange segments at ${r}_{max}=1.0$ form two corresponding discontinuous structural units.

To understand how chromatin is structured and packaged at a local level, we developed a bottom-up approach to identify localized structural units beginning with intra-chromosomal Hi-C data at 5 kbp resolution. We quantify the degree of association between two structural units $i$ and $j$ by the ratio ${r}_{ij}$ of the interaction energy (given by total Hi-C interaction counts) between units $i$ and $j$, denoted ${F}_{ij}$, to the geometric average of interaction energy within the individual units (ignoring self-interactions at the 5 kb level), denoted ${F}_{i}, {F}_{j}$ respectively: ${r}_{ij}={F}_{ij}/\sqrt{{F}_{i}{F}_{j}}$. The interaction energy is measured in number of contacts detected in Hi-C experiment between corresponding structural units. The procedure begins by considering each 5 kb bin as a separate structural unit, and iteratively merges pairs of structural units with the highest value of ${r}_{ij}$. The global maximum value of ${r}_{ij}$, denoted ${r}_{max}$, decreases monotonically with each iteration, and at lowest ${r}_{max}=1.0$ the structural units of first level of hierarchy are obtained. Continuing with the iterative procedure reduces ${r}_{max}$, yielding larger structural units that are more physically separated at lowest ${r}_{max}=\left[0.8, 1\right), [0.6, 0.8)$, and [0.5, 0.6), revealing the hierarchical organization of chromatin structure. The result of applying the procedure on a 1 Mb region is represented schematically in Fig. 3 (see also Supplementary Figure S6), where the top shows 1D representations of the structural units at different levels, and the bottom shows how individual 5 kb bins (black nodes) are grouped into structural units at different levels of hierarchy (grey, blue, red, and green ellipses). Supplementary Table S2 lists the average unit size at lowest ${r}_{max}=1.0, 0.8, 0.6, 0.5$ for chromosomes 1, 11, and 14 in each of the samples c152, c187, p102, and p146. For the sake of definition from now on, the ${r}_{max}=1.0, 0.8, 0.6, 0.5$ designate ${r}_{max}\ge 1$, $\left[0.8, 1\right), [0.6, 0.8)$, and [0.5, 0.6), respectively. The scripts developed for and used in the local structural unit analysis are provided in Bitbucket (bitbucket.org/ZhenWahTan/chromatin-localunitanalysis/). The scripts for the 3D chromatin ensemble reconstruction (The Python package ChromaSEP developed in^32^) are also provided in BitBucket (URL: https://bitbucket.org/ZhenWahTan/chromasep/).

### Ethical approval

No experimental animals or human participants were used in the study. All the data used in the study were obtained from public resources associated with previously published research – see below for the data sources and accession numbers.

## Results

### Reorganization of chromosomal territories in OSNs of SARS-CoV-2-infected patients

We use here recently developed two-step procedure consisting of Markov state modelling for identifying structural units and stochastic embedding procedure for obtaining the 3D ensemble reconstruction of the whole-genome chromatin structure, elucidating morphology of chromosomal territories (CTs) and intermingling between them. Inspecting obtained ensemble reconstructions of OSN chromatin visually (top rows in Fig. 1 and Supplementary Figure S1) we found that although the overall organization of chromosomal territories (CTs) shows some degree of similarity across all samples, patient samples yield noticeable shifts and diversity in CT positioning, morphology, and their intermingling. Non-cell-autonomous disruption of nuclear architecture that results in a damage of genomic compartments containing OR genes was shown as a potential cause of COVID-19-induced anosmia in both hamsters and humans^20^. Mouse studies have indicated that gene regulation in OSNs depends upon inter-chromosomal interactions between key regions^30,31^: given that the human homologs of these genomic regions are also distributed across different chromosomes, it is likely that similar inter-chromosomal interactions may also be critical for OSN gene expression regulation in humans. One major class of OSN-associated genes are the olfactory receptor genes (ORs), which bind odorants in the nasal passage and enable odor perception and discrimination. OR genes are distributed across 17 somatic chromosomes in humans, of which the largest sets are located on chromosomes 11 (166 OR genes), 1 (62), 9 (25), 14 (23), 19 (20), and 12 (17) — the centers of these chromosomes are labelled in cross-sectional views below the full reconstructions in Fig. 1 (a larger, equally-spaced series of cross-sectional views is given in Supplementary Figure S1). We observed that chromosomes 9, 14, and 19 were consistently located near the center, chromosomes 12 and 11—near the periphery in all 5 samples, while chromosome 1 shifted towards the nuclear periphery in infected patients. Comparing the radial distribution of CTs (right panel of Fig. 1) confirms that, indeed, while the radial positioning of most chromosomes remained unchanged between controls and patients, chromosome 1 was noticeably displaced outwards in OSNs of infected patients. Kruskal–Wallis test shows highest difference in positioning of chromosome 1 in controls and patients is H = 4.2e4, *p* < 1.0e − 300. For the other key chromosomes mentioned, the H statistic are all smaller, the largest being chromosome 14 with H = 1.0e4 (*p* < 1.0e − 300): chromosome 9 (H = 3.2e3, *p* < 1.0e − 300), chromosome 11 (H = 0.7e3, *p* < 4.79e − 159), chromosome 12 (H = 1.7e3, *p* < 1.0e − 300), chromosome 19 (H = 8.6.0e3, *p* < 1.0e − 300). The complete data on the differences between locations of chromosomes in control and patient reconstructed chromatin structures is provided in Supplementary Table S3.

To systematically quantify and compare CT organization, we identified chromosome pairs that showed consistent and strong intermingling in either controls or patients. In this work, chromosome $i$ is defined as strongly intermingling with chromosome $j$ if the mixing fraction ${m}_{ij}$ exceeds a threshold value of 10% (see “Materials and methods” section). Our results are summarized by the network in Fig. 2A, where grey arrows indicate strong intermingling present in all 5 samples, blue/red arrows indicate strong intermingling in all control/patient samples, respectively (see Supplementary Figure S4 for separate intermingling networks for each sample). Two groups of intermingling CTs can be identified in all samples, demarcated by dashed outlines in Fig. 2. Firstly, chromosomal group A comprises mainly of smaller chromosomes, with four of the acrocentric chromosomes^13,14,21,22^ showing exceptionally strong intermingling in both controls and patients. The data also showed chromosome 9 to be a central component of the structure of group A in controls, forming strong intermingling with almost all chromosomes in the group. However, many of these structural links were lost in the patient sample p102, resulting in a sparser network of CT intermingling in group A for patients. Secondly, chromosomal group B consists of larger chromosomes forming highly consistent and strong intermingling in all samples, with slightly stronger intermingling overall in patients. Control samples also formed a smaller, third chromosomal group C, where chromosomes 12 and 19 intermingled strongly with chromosome 1. The decoupling of chromosome 1 is especially evident in patient samples p147 and p102, due to the displacement of chromosome 1 towards the periphery as observed above (Fig. 1).

Since many structural differences between controls and patients involved chromosomes with many OR genes, we sought to also understand detailed changes in CT morphology by studying how one-chromosome and two-chromosome distance correlation functions (see Fig. 2B and Supplementary Figure S5) are different between controls and patients, focusing on c187, p102, and p147 as representative examples. The distance correlation function $g(r)$ is a quantitative description of the morphology of non-rigid structures, and it is defined as the probability distribution of distances between any two randomly selected points in the structure. Typically, CTs form an approximately globular structure (typically crumpled or fractal globule^39^) that has the distance correlation $g(r)$ forming a single peak at a position corresponding to the size of the globule: for example, in c187, chromosomes 1 and 19 have sizes of about 8 and 4 a.u., respectively (Fig. 2B). Long tails in the distribution signal a deviation from sphericity, typically due to diffuse boundaries or elongated protrusions from the structure, such as the case of chromosome 1 in p102 and p147 (Fig. 2B). Two-chromosome distance correlations can exhibit a range of functional forms depending on the degree of intermingling between the chromosomes. On one end of the spectrum, for chromosome pair 1–19 in p147 we observed a well-separated double peak structure in $g(r)$, which indicates a clear separation of chromosomal territories—the first peak at 8 a.u. is the average size of individual CTs, and the second peak at 25 a.u. indicates the separation between the CT centers. Towards the other end of the spectrum, in c187 we observed that the $g(r)$ curve for chromosome pair 1–19 contains only one dominant peak at 8 a.u., indicating strong intermingling between the two CTs. Comparing the structures and $g(r)$ plots for chromosome pairs 1–12 and 1–19, it becomes evident how the consistent, strong intermingling between these chromosome pairs in control OSN chromatin (exemplified by c187 in Fig. 2B) was weakened or lost in infected patients (e.g., p102 and p147 in Fig. 2B), potentially impacting processes that depend on inter-chromosomal interactions within chromosomal group C. Turning to chromosomal group A, where we observed the weakened intermingling of chromosome 9 with many other CTs in the group, we focused on the chromosome pair 9–14, both of which contain a large number of OR genes. In c187, we observed that the two-chromosome $g(r)$ is virtually indistinguishable from the one-chromosome $g(r)$ for the larger chromosome 9, indicating exceptionally strong intermingling between these CTs. Reconstructions of patient OSN chromatin, however, showed more diversity in CT morphology and intermingling. In p102, the distinct double peak structure in $g(r)$ indicates a clear separation of CTs with minimal intermingling despite having a shared boundary. On the other hand, in p147, chromosome 14 formed a more dispersed CT that overlaps significantly with chromosome 9. The structure of chromosome 14 in p146 (see Supplementary File, Figure S3) represents an intermediate state between p102 and p147, where a poorly resolved secondary peak in pair-wise $g(r)$ indicates a moderate degree of intermingling between the territories of chromosomes 9 and 14. Several cases of chromosomal intermingling consistently seen in chromatin of OSNs in control subjects were also altered to varying degrees in chromatin of OSNs in infected patients. We have performed Kolmogorov–Smirnov test of difference between selected two-chromosome distance correlation function and the one-chromosome distance correlation function of the larger of the two chromosomes in Fig. 2. The test shows weakening of the intermingling between chromosomes in patients compared to controls in most of the cases (Supplementary Table S4). It can be speculated that different trend for intermingling of chromosomes 9 and 14 in patient 147 case is associated with non-cell-autonomous nature of observed disruptions of the structural nuclear architecture upon COVID-19 infection^20^. At the same time, it might be an artefact of the limited sample data, which should be further investigated when more data will become available. As many of these changes involve chromosomes containing a large number of OR genes, the altered structures of the former may interfere with and/or disrupt key processes dependent on the presence and regulation of key inter-chromosomal contacts^30^.

### Analyzing the relationship between fine structure and regulation of olfactory receptor clusters

Direct experimental data on OR gene expression and regulation in human OSNs became available within the last few years. Downregulation of OR genes as a result of the non-cell-autonomous disruption of interchromosomal OR compartments in human was recently shown^20^. While it was not directly established that downregulation in OR signalling genes causes anosmia upon COVID-19 infection, authors inferred it based on the phenotypes of knockout mice^20^. The mice are a well-established model organism for the olfactory system with corresponding functional and structural patterns. All the above prompted us to explore regulation mechanisms in human samples based on 3D whole-genome reconstruction of the chromatin ensemble and using local analysis of the chromatin structural hierarchy in OR-containing regions. Current data on OR gene regulation in mouse OSNs point to (i) the heterochromatic compaction of OR gene clusters^26,28^, and (ii) activation of selected OR genes via looping interactions with enhancers, dubbed the Greek Islands, that are located near corresponding OR gene clusters^29,31^. Understanding how altered chromatin structure affects OR gene expression would therefore require us not only to zoom in on a finer genomic scale, but also to consider interactions of OR genes with potential enhancers located near corresponding gene clusters. We postulated that since many mouse transcription factors have orthologs in humans^40^, enhancers in mice may also show some degree of conservation in humans, hence a direct mapping of the Greek Island enhancers found in mice onto the human genome may provide a reasonable set of putative enhancers (PEs) of OR genes in human OSN chromatin. We mapped the 35 Greek Islands enhancers from the mouse genome to the human genome, identifying a subset of 15 PEs located within 1 Mb of at least one human OR genes (mapping results are listed in Supplementary Table S1). Noteworthy, PEs considered in this work include: (i) Milos, located < 200 kbp from the only cluster of Class I OR genes located in chromosome 11, (ii) Symi, Lesvos, Skiathos, and H, located on the flanks of two Class II OR gene clusters in chromosome 14, and (iii) Thira, located within a large Class II OR gene cluster near the q-arm telomere of chromosome 1. Having obtained the genomic coordinates of OR genes and their putative enhancers, we then developed an analytical procedure using Hi-C interaction data to identify strongly localized structural units and the interaction energies between them, enabling us to investigate if human OR genes are also physically compacted, and if these genes form preferential interactions with the putative enhancers identified above. Figure 3 (see also Supplementary Figure S6) shows a schematic diagram illustrating this procedure performed on the genomic region chr11:5.5–6.5 Mb: using the Hi-C interaction matrix at 5 kb resolution. We first considered each 5 kb bin as an individual structural unit, represented by black circular nodes in the network schematic at the bottom of Fig. 3, joined by a curve representing the chromosomal trace. We then iteratively merged pairs of structural units with the highest interaction energy ratios $r$, defined as the ratio of interaction energy between pairs of distinct units to the average interaction energy within the units. The highest value of $r$ in the remaining network, ${r}_{max}$ decreases monotonically, and when ${r}_{max}$ falls below 1.0 we obtain the set of structural units marked by grey circles in the network. A 1D genomic representation of the structural units is shown at the top of Fig. 3: white-colored horizontal bars indicate a structural unit made up of a contiguous series of 5 kb bins, while colored bars of the same color indicate non-contiguous segments that join to form a single structural unit at the given level. Decreasing the value of ${r}_{max}$ by further merging of units leads to a greater degree of separation between larger structural units: the blue, red, and green ellipses in Fig. 3 and Supplementary Figure S6 correspond to structural units obtained with the lowest ${r}_{max}=0.8, 0.6, 0.5$ (see “Materials and methods” section), respectively, indicating the presence of a hierarchy in chromatin structure at corresponding scales. In this work, to obtain a sufficient degree of physical separation between structural units in the analysis of differences in patient and control samples we used the cutoff ${r}_{max}=0.6$. In general, we observed that patient samples had larger interaction patterns within each chromosome, leading to larger in sizes structural units compared to control samples (see Supplementary Table S2). The structural units obtained for ${r}_{max}=0.6$ and $0.5$ have typical size of (sub)TADs with 100–400 kb sizes, apparently demarcating continuous and discontinuous TADs^37^ containing ORs and their regulatory elements. The higher ${r}_{max}=0.8$. and $1.0$ allow to delineate other basic structural units of chromatin, loops^34–36^ with characteristic sizes of 30–60 kb, forming the (sub)TADs and allowing to explore the structure and interactions in the latter and their alterations between normal and pathological states.

**Figure 4:**
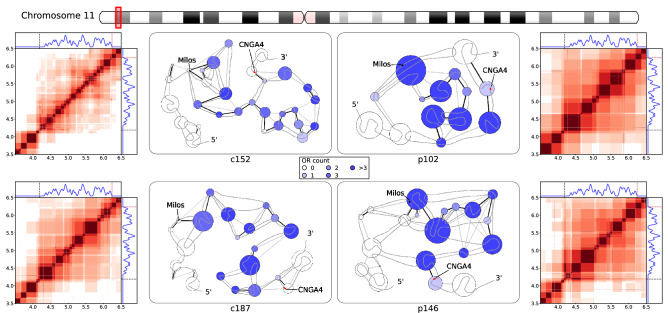
Fine structural organization in the Class I olfactory receptor gene cluster on chromosome 11. Left and right columns. Heatmaps of interactions between structural units in the region 3.5–6.5 Mb of chromosome 11. In the heatmaps, dark red squares along the diagonal mark the size and position of continuous structural units, and off-diagonal colored regions indicate strongly-interacting unit pairs ($r>0.05$). The adjoining panels (above and to the right of the heatmaps) show the 1D distribution of Class I olfactory receptor genes (blue trace), putative enhancers mapped from mouse annotations (black dashed lines), and human mature OSN marker genes (red dashed line). The heatmaps for control samples c152 and c187 are shown on the left column, while patient samples p102 and p146 are shown on the right column. Center. Network representations of chromatin structure in the same region 3.5–6.5 Mb of chromosome 11, including the PE Milos and the OSN marker CNGA4. Structural units are represented as circles of radii scaling with unit sizes, colored by the number of ORs in the unit. Black/grey edges connecting the units indicate strong interactions, with black edges indicating an interaction energy ratio $r>0.5$ and grey edges indicate $0.2<r\le 0.5.$ A black trace is overlaid on the network to show genomic positions of the units in the 5’-to-3’ direction on the positive strand. The approximate genomic locations of the PE Milos and the OSN marker CNGA4 are represented by black and red marks respectively.

We began by studying the organization of the only cluster of 54 Class I OR genes in the region 3.5–6.5 Mb of chromosome 11, flanked by a PE Milos and an OSN marker gene CNGA4. Here and below and we perform our analysis on two controls and two patient subjects. Aggregate metrics comparing the sizes and interactions between OR gene-containing (ORc) and OR gene-free (ORf) units are provided in Table 1. In the interaction heatmaps for each sample (Fig. 4), the position and size of structural units are indicated by dark red squares along the diagonal, while strong interactions (with interaction energy ratio $r>0.05$) between units are marked by colored off-diagonal areas. The interaction energy between units is measured in number of contacts detected in Hi-C experiment between corresponding units. Adjoined to the interaction heatmaps are two panels that depict the density profile of OR genes (blue plot), while the location of PE Milos and the OSN marker gene CNGA4 are marked by black and red dashed lines, respectively. More structural details are illustrated in the network diagrams (Fig. 4), where we represented structural units by circles with radii corresponding to unit sizes. Strongly interacting units are joined by black/grey edges: black edges indicate an interaction energy ratio strong interaction with $r>0.5$, grey edges indicate interactions with $0.2<r\le 0.5$, and weaker interactions are omitted for clarity. The grey curve serves as a chromosomal trace in the 5’-to-3’ direction. The distribution of OR genes across structural units is represented by the color saturation of the units, and the location of the PE and the OSN marker gene are represented schematically by the black and red boxes along the chromosomal trace. Beginning with aggregate statistics (Table 1), we observed that in this region, in comparison with control samples, all patient samples formed larger units overall. Also, the average interaction energy ratios $r$ between structural units were higher in patient than in controls, manifesting stronger packing within these units. The Orc units show stronger difference between the patients and controls than ORf ones (Table 1), pointing to a consistent change in chromatin architecture between controls and patients. At the same time, we observed that given higher or comparable compactness (Table 1), the interactions between OR gene-containing units were substantially stronger than that of OR gene-free units in all samples (Table 1), suggesting that the OR gene cluster exhibits more compact packing than regions without OR genes – consistent with the current view (based on mouse models) of OR gene compaction and silencing in heterochromatin.

**Table 1:** Characteristics of structural units identified in neighborhoods of olfactory receptor gene clusters in chromosomes 1, 11, and 14. The average size of structural units and the average interaction energy ratio between structural units in selected regions containing OR clusters, featured in Figs. 4,5,6. The average size and interaction energy ratios for subgroups of OR-containing (ORc) and OR-free (ORf) structural units were also included, along with the ratio of averages for ORc to ORf subgroups.

Location	OR Class	Sample	Average structural unit size				Average interaction energy ratio			
			Overall	ORc	ORf	ORc/ORf	Overall	ORc	ORf	ORc/ORf
Chromosome 11 3.5–6.5 Mb	I	c152	103.7	97.9	113.6	0.86	0.115	0.164	0.108	1.52
		c187	144.8	147.1	141.1	1.04	0.125	0.197	0.150	1.31
		p102	235.4	206.4	341.7	0.60	0.197	0.254	0.178	1.43
		p146	165.0	170.4	156.9	1.09	0.153	0.248	0.170	1.46
Chromosome 1 246–249 Mb	II	c152	101.0	80.3	135.0	0.59	0.093	0.150	0.138	1.09
		c187	94.7	82.6	107.5	0.77	0.107	0.168	0.164	1.02
		p102	163.9	119.6	212.3	0.56	0.131	0.207	0.184	1.13
		p146	141.7	98.9	191.7	0.52	0.121	0.193	0.119	1.62
Chromosome 14 19.5–22 Mb	II	c152	95.4	72.1	112.8	0.64	0.091	0.133	0.107	1.24
		c187	103.5	86.4	118.1	0.73	0.100	0.118	0.093	1.27
		p102	134.2	131.1	137.0	0.96	0.127	0.175	0.101	1.73
		p146	121.1	146.8	95.5	1.54	0.098	0.158	0.090	1.76

Further features of the organization of the OR gene cluster can be seen in the interaction heatmaps (see Fig. 4). In controls there is a clear division of the OR gene cluster into 3 strongly interacting subsections (approximately 4.2–5.1 Mb, 5.1–6.0 Mb, and 6.0–6.5 Mb) with relatively weak interactions between them. In patients, however, strong long-range interactions within the OR gene cluster render these subsections barely distinguishable, in agreement with the hypothesis that the OR gene cluster is more compacted in patients. The network representation of structural units shows further details on the structural environment of key genomic elements: while control samples had the PE Milos located in a small unit free from OR genes, in both patient samples we found that the PE Milos formed part of a large structural unit containing multiple OR gene clusters (450 kb unit with 5 ORs in p102, 300 kb unit with 6 ORs in p146). Also, for controls the OSN marker gene CNGA4 (44 kb downstream of the nearest OR gene) resided in ORf units, but for patients the marker gene was included in the same structural unit as the proximal OR gene. Taken together with the observation of strengthened OR gene cluster compaction in patients, and the model of heterochromatic silencing on compacted OR genes in mouse models, the inclusion of the PE Milos and OSN marker CNGA4 in ORc structural units may result in the silencing of both genomic elements. Besides the direct impact of CNGA4 downregulation, the inactivation of the PE Milos may also have indirect consequences on OSN function. Given that Class I OR genes have been assumed to rely primarily on intra-chromosomal interactions for activation, the silencing of the PE Milos may significantly impair the transcription of Class I OR genes if no other enhancers are located within the vicinity—the next closest putative enhancer, *P*, is located 2.7 Mb downstream, more than 600 kb from the other end of the OR gene cluster.

**Figure 5:**
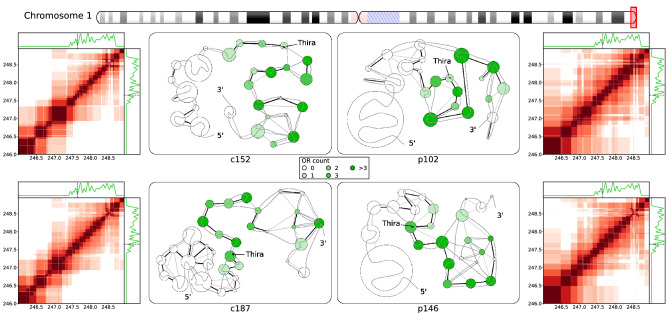
Fine structural organization in a Class II olfactory receptor gene cluster near the chromosome 1 q-ter telomere. Notations and marks are as in Fig. 4. Heatmaps of interactions and network representation are given for structural units in the 246.00–248.94 Mb region of chromosome 1. Structural units are indicated by dark red squares along the diagonal, and off-diagonal-colored regions show interactions with energy ratios above a threshold, $r>0.05.$ The distribution of Class II OR genes is indicated by the adjoining 1D plots by the green traces, and the location of the PE Thira is marked by the black dashed line. Network representation of structural units in the OR cluster, with strong interactions indicated by black/grey edges, and the approximate location of the PE marked by a black mark.

Turning to the Class II OR genes scattered across the human genome, we observed that these gene clusters show rather consistent trends in structural organization, which is exemplified here with the large cluster of 43 Class II OR genes 246.0–248.9 Mb in chromosome 1. Table 1 indicates that OR gene-containing (ORc) structural units are smaller in size (0.52–0.77) than OR gene-free (ORf) units in this region. Interactions in ORc units are stronger than that in ORf units, and patient samples show a greater enrichment of interactions in general. Figure 5 shows the interaction heatmaps and network representations of structure, in the distribution of OR genes denoted by the green density plot and color saturation in structural units and a single PE Thira identified in this region. Comparing the interaction heatmaps for controls and patients, we observed firstly that the region upstream of the OR gene cluster (approximately 246.0–247.5 Mb) showed little interaction with the OR gene cluster in controls, but in patients this region tends to interact more strongly with the whole OR gene cluster. Also, the telomeric region (248.8–248.9 Mb) interacts with both ends of the OR gene cluster more strongly in patients (with $r\approx 0.10$) than in controls ($r<0.05$). The network representations illustrates that the final ~ 300 kb of the OR gene cluster (248.4–248.7 Mb) forms a set of structural units comprising interspersed genomic segments, indicating a high degree of complexity in chromatin organization in that region. In the region 247.5–248.4 Mb, control samples yield a series of weakly-interacting, small structural units, whereas in patients a series of small but strongly interacting genomic units in this region (observed in p102) or large units with weak long-range interactions between them (observed in p146). In comparison, control samples adopt a relatively sparse conformation in this section, forming a series of small units interact strongly only with adjacent units. This picture indicates a greater degree of physical compaction of the OR gene cluster in patients compared to controls, in agreement with the observations made about the Class I OR gene cluster. Together with the stronger interactions between the OR gene cluster and the telomeric region, above observations may suggest that the increased physical compaction of this region in patients may be linked to the “spread” of telomeric heterochromatin. Surprisingly, the only regulatory element located in this region, the PE Thira, was located within OR gene-containing units in both the controls and patients. While physical compaction of the OR gene cluster may lead to inactivation of this PE, work on mouse models indicate that inter-chromosomal enhancer-promoter interactions play a key role in regulating Class II OR genes^31^, potentially availing other PEs for OR gene expression in this cluster. As the available Hi-C dataset is insufficient for studying inter-chromosomal interactions robustly at this resolution, further work is required to investigate how changes in inter-chromosomal interactions may affect regulation of Class II OR genes. Further assays would also be required to identify regulatory elements human ORs to elucidate epigenetic mechanisms of Class II OR genes in normal and infected human OSNs. Considering examples of observed changes for Class I and II OR genes upon COVID-19 infection (Figs. 4 and 5, respectively), one may infer some mechanisms and features of the OR gene regulation. For example, in both cases downregulation of OR gene is facilitated by stronger packing of chromatin regions. At the same, while the Class I OR genes prone to formation of larger and denser local clusters, regulation of those belonging to Class II is apparently also facilitated by the packing interactions with heterochromatin: centromeric and telomeric in case of chromosomes 1 and 14 considered in this work, respectively. Supplementary F7 exemplifies the resulting difference in the picture of nuclear architecture changes observed for Class I (chromosome 1) and II (chromosome 11) OR genes-containing regions.

**Figure 6:**
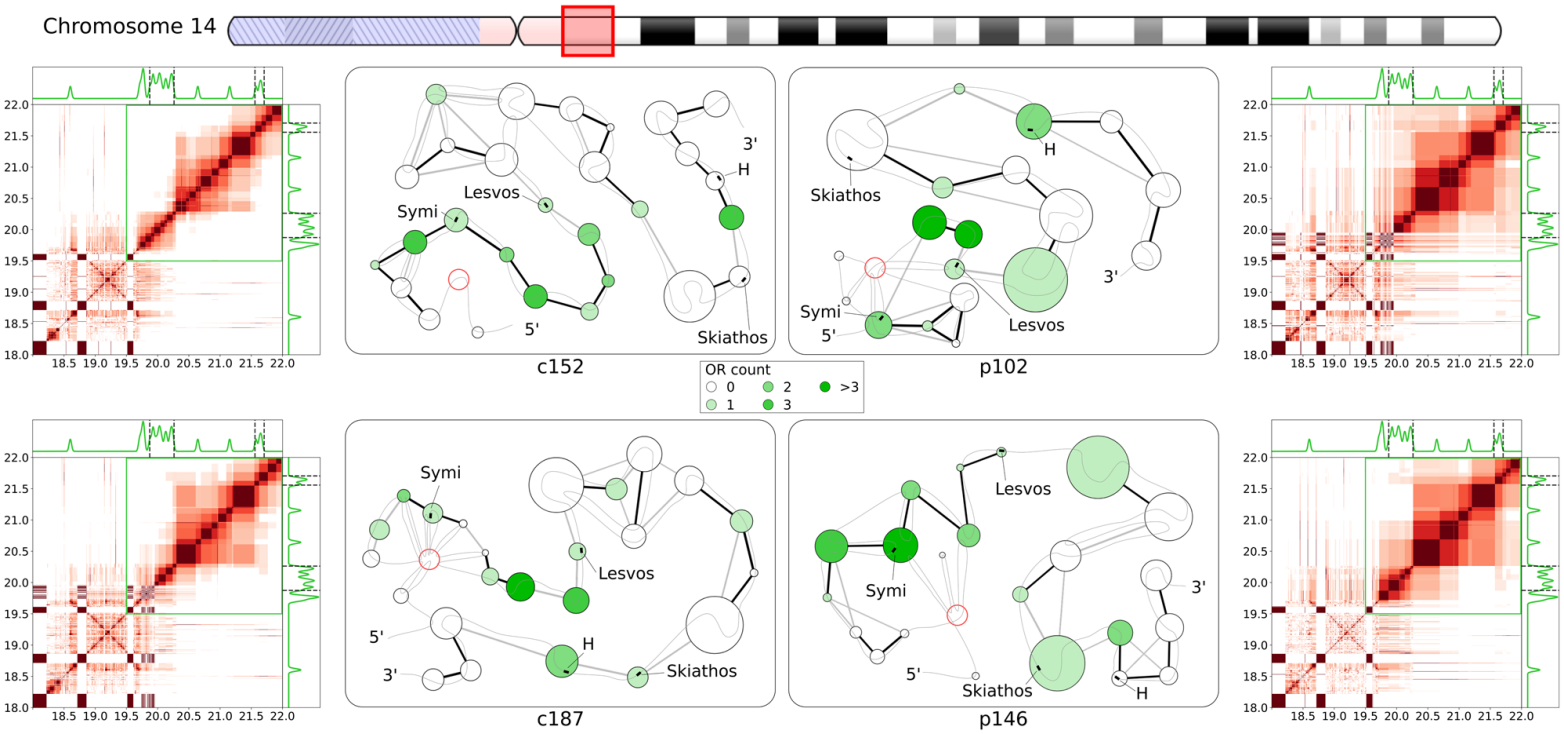
Fine structural organization in two Class II olfactory receptor gene clusters near the chromosome 14 centromere. Notations and marks are as in Fig. 4. Heatmaps of interactions and network representation are given for structural units in the region 18.0–22.0 Mb of chromosome 14. Two OR clusters with nearby PEs are present in this region: the cluster at 19.8–20.3 Mb contain PEs Symi and Lesvos, while the smaller cluster 21.5–21.7 Mb is flanked by PEs Skiathos and H. In the network representation the large structural unit associated with pericentromeric heterochromatin is composed of multiple non-contiguous segments, and it is represented here by the single node with a red border.

A further example of Class II OR gene clusters can be found in the pericentromeric region of chromosome 14, around the coordinates 19.5–22.0 Mb, with 4 PEs mapped into the region. This region comprises two separate clusters, with a larger one (19.8–20.3 Mb) containing 17 OR genes, flanked by the PEs Symi and Lesvos, and a smaller one (21.5–21.7 Mb) containing 3 OR genes, flanked by PEs H and Skiathos. Table 1 shows that patients reveal larger structural units than controls as well as stronger interactions in them, which is chiefly result of stronger interactions in ORc units. Figure 6 provides further details, with the interaction heatmap zoomed out to include the pericentromeric region from 18.0 Mb. The green frame marks out the region 19.5–22.0 Mb that we focus on in the network representation, where we included a red node to represent the large, multi-segment unit associated with centromeric heterochromatin. The interaction heatmaps for controls show a separation of the upstream cluster from the downstream ORf region, while in patients we observed stronger interactions between the two. The OR gene cluster also tends to form sparser interactions in controls than in patients, concurring with our general observations in Table 1. The network representation shows that the upstream OR gene cluster in controls formed only a series of small units in a loosely packed conformation, yielding strong interactions only with adjacent units (despite small segments grouped with centromeric heterochromatin in c187) similar to structural units and interactions between them in controls of the Class II OR gene cluster in Fig. 5. In patient samples the upstream cluster formed a set of highly interlinked units with centromeric heterochromatin, while downstream region reveals formation of larger structural units, much like that previously observed in chr1:248.4–248.7 Mb. Shifting our focus to PEs, while Symi and Lesvos were consistently located in ORc units for all samples, there is a movement of H and Skiathos between ORc/fr units with no consistent difference observed between patients and controls. Notably, all PEs are observed in larger structural units in patients compared to controls.

Summarizing the analysis of the internal organization of OR gene clusters by identifying fine structural units using high-resolution Hi-C data and by quantifying the interaction energy between these units, we obtained the several general observations: In most of the cases the average size of ORc structural units is smaller than that of ORf ones. The ORc units also form consistently stronger mutual interactions than ORf counterparts in the same regions, with a stronger bias seen for patients in Class II OR gene clusters (Table 1). Patient samples generally form larger structural units and stronger interactions. The interactions in ORc units are always stronger than in ORf ones in both controls and patients with more pronounced effect in the latter. Network representations of structural units in OR gene clusters illustrate less compact, more linear organization of ORc units in controls, while patients either formed larger units overall, or formed a series of small units with complex interlinking or strong interactions between them (Figs. 4,5,6 and Supplementary Figure S7). These observations suggest stronger physical compaction and potential involvement in silencing interactions of OR gene clusters in pathology, which can disrupt OR gene expression leading to anosmia in COVID-19 patients. The increased structural units and their compaction can be a general mechanism of OR silencing. For example, and the grouping of the upstream PE Milos and the downstream OSN marker gene CNGA4 in the ORc units in patients in the Class I OR gene cluster (Fig. 4) in large structural units with increased packing density may work as a mechanism by which olfaction may be impaired in COVID-19 infected patients. In case of Class II OR gene clusters higher compactness yielded upon silencing (examples considered in this work also point to the role of heterochromatin, telomeric/centromeric in case of chromosome 1/14, see Figs. 5 and 6, and Supplementary Figure S7, facilitating the packing) may prevent formation of interchromosomal cluster typical for actively expressed Class II OR genes.

## Discussion

While anosmia has been a useful diagnostic indicator for early-stage COVID-19 infection (1–4), mechanisms behind its rapid onset and recovery has been a subject of debate. As human olfactory sensory neurons (OSNs) do not express the primary receptors that enable direct infection by the SARS-CoV-2 virus, it has been proposed that inflammatory response and damage to support cells in the olfactory epithelium (OE) may result in olfactory impairment^8–11^. Furthermore, as most patients recover their sense of smell within a short time frame of 1–2 weeks^12–15^ it is unlikely that these changes have led to massive cell death among OSN populations, thus we set out to understand how COVID-19 infection in the OE may affect olfactory signal transduction in OSNs non-cell autonomously. Given that olfactory receptors (ORs) are vital in initiating olfactory signal transduction in OSNs, a recent study linking inflammatory cytokine signaling to decreased OR gene expression^22^, where upregulation of certain interferon regulated genes led to chromatin reorganization because of the immune response, prompted us to investigate how OR gene regulation is determined and modulated by chromatin organization. Recently published work^20^ showed that non-cell-autonomous reorganization of the neuronal nuclear architecture can potentially affect genomic compartments containing OR genes. While downregulation of OR/OR-signalling genes was not directly linked to an emergence of the COVID-19-induced anosmia in this work, authors inferred this connection from the phenotypes of knockout mice^20^. Considering earlier observations on OR gene regulation in mouse OSN chromatin structure^27,28,31^ supported by the above work^20^, we aimed here at the study of structural foundation of the OR gene function disruption that can be caused by chromatin reorganization taking place upon SARS-CoV-2 infection. Specifically, we used Hi-C data on human OSNs to study if features identified in mouse models have identifiable parallels in humans, such as the interchromosomal aggregation of OR genes^27,38^ and looping interactions between OR gene clusters and nearby enhancers^29^, and if these features are disrupted upon COVID-19 infection in the OE. To achieve our goal, we first reconstructed whole-genome chromatin ensembles using a stochastic embedding procedure we have previously developed^32^. While the relative positioning of chromosomal territories (CTs) in SARS-CoV-2-infected patients and control subjects were somewhat similar (see Fig. 1), we observed that chromosomes containing the most OR genes tend to intermingle strongly, consistent with the interchromosomal OR gene aggregation observed in mice^28^. This intermingling was significantly weakened in infected patients: notably, chromosomes 1 and 9 showed significant detachment from all other chromosomes in patient samples. While having chromosomal intermingling cut off may hinder the formation of interchromosomal enhancer hubs^30,31^, understanding how structural features of chromatin relate to OR gene regulation required us to probe finer levels of detail, looking at chromatin architecture at the level of OR gene clusters and putative enhancers (PE) mapped from mouse data. To this end, we developed here an analytical method for identifying local structural units down to an average size of (sub)TADs^41^, 100–200 kb and further down to forming TADs chromatin loops with characteristic sizes of 30–60 kb in order to explore structural units and interactions between them relevant to OR gene regulation. We observed that OR gene-containing structural units had consistently stronger mutual interactions than OR gene-free units in all samples, which is indicative of the heterochromatic-driven physical compaction of OR genes observed experimentally^27,28^. Interestingly, this effect was more pronounced for patients in Class II OR gene clusters (Table 1). The stronger interactions observed between OR gene-containing (ORc) units leading to formation of larger structural units are more pronounced in patients (Figs. 4,5,6), pointing to a potential disruption of chromatin organization within OR gene clusters for COVID-19 patients. For example, we observed that key genomic elements near the Class I OR gene cluster (Fig. 4), including the only PE (Milos) in that region and a marker gene critical for OSN function (CNGA4), were grouped into larger in sizes and more densely packed ORc structural units in patients (Table 1), pointing to a potential intrachromosomal condensation as a mechanism of the Class I OR genes downregulation that potentially leads to olfactory dysregulation. According to previous works, the structural context of OR genes and their regulatory elements in Class II OR gene clusters yield multiple contacts with heterochromatin of telomeric and centromeric regions in chromosomes 1 and 14, respectively, studied here. These interactions may interfere with the formation of interchromosomal clusters, which were shown as important element of regulation in case of Class II OR genes. Noteworthy, the mapping of putative enhancers was performed in this work on the basis of mouse data^30,31^, relying on the evolution-based hypothesis about similarity between clusters of genes performing same functions. It makes conclusions of this work to be rather descriptive and not having strong predictive power. At the same time, observations made here can be used as a starting point to future work, providing a multi-scale picture of the structural foundation for OR genes regulation from the level of chromosome intermingling and forming functional gene clusters to the regulation of individual genes in (sub)TAD chromatin regions.

Overall, the major goal of this work was to explore anticipated structural basis of the COVID-19-induced anosmia. While recent experimental work showed that SARS-Cov-2 infection of human olfactory epithelium (OE) coincides with downregulation if OR/OR signalling genes via disruption of genomic compartments^20^, no description of structural specifics underlying this observation was provided. In this work, using our unique methodology of the whole-genome chromatin ensemble reconstruction^32,33^, we obtained here first global picture of structural changes in the architecture of reconstructed 3D chromatin in COVID-19 patients. Analyzing fine, high-resolution structural hierarchy of the OR-containing genes chromatin regions using a new method developed in this work (Figure and Supplementary Figure S6) we also obtained some details of potential changes in local intrachromosomal packing and their possible role in modifying interchromosomal OR genes-containing clusters. Despite many open questions about functional significance of structural details in OR gene clusters due to insufficient data, our analysis here indicates significant alterations on both inter- and intrachromosomal levels in the chromatin of the COVID-19 patients. Specifically, in terms of the structural environment of chromosomal territories, the chromosomes enriched with ORs genes showed significant changes in the degree of intermingling in SARS-CoV-2-infected patients. Together with additional intrachromosomal interactions of OR-containing region with telomeric and centromeric heterochromatin in chromosomes 1 and 14, respectively, it can be involved into disruption of interchromosomal clusters of the Class II OR genes resulting in their COVID-19-induced inactivation. The intrachromosomal structural hierarchy in the vicinity of OR genes and enhancers shows the formation of (sub)TADs from chromosomal loops and alteration of their structure in pathological samples that may contribute to the onset of anosmia in COVID-19 patients. In particular, enlarged structural units with higher density of interactions observed here in chromosome 11 may serve as an example of the Class I OR genes downregulation mechanism.

Given the importance of understanding molecular mechanisms of SARS-CoV-2 infection for future development of sensitive diagnostics and drugs for emerging new variants of concern (VOCs), the genomic and epigenomic studies should be further extended and developed into more precise approaches with predictive and design capabilities^42^. These tasks will require a number of improvements in resolution of experimental techniques and additional data on epigenetic factors and their mapping on genomic sequences: (i) it should include further development of Hi-C analysis and relevant techniques^43–45^ for mapping epigenetic signals, their merging in combined pipelines^46,47^, and the consideration of chromatin-lamina interactions in the reconstruction of 3D chromatin structure, to name a few; (ii) currently missing information on relevant regulatory elements and epigenetic signals^48,49^ should also be obtained and mapped on genomic sequences to obtain a complete picture of genome expression and regulation in norm and pathology. Finally, developed here methodology for the analysis of high-resolution structural hierarchy with (sub)TAD-size units in combination with the 3D ensemble reconstruction of the whole-genome chromatin^32^ can be instrumental in future studies of the role of chromatin structure and dynamics in normal and pathological developments with anticipated implications for diagnostics and treatments. It would be, however, of utmost importance to directly link above methods to experimental efforts, to calibrate and develop them based on clinical and experimental data, and to eventually build a combined theoretical–experimental research framework.

## Supplementary Information

Supplementary Information.

## Data Availability

The datasets used and/or analysed during the current study available from the corresponding author on reasonable request.
