# Supplementary material for: Disrupted chromatin architecture in olfactory sensory neurons: looking for the link from COVID-19 infection to anosmia
Source: Sci Rep. 2023 Apr 11;13:5906. doi: 10.1038/s41598-023-32896-8 (PMC10088727; doi:10.1038/s41598-023-32896-8)
Supplement: Supplementary file 1 — Supplementary Information. [file 41598_2023_32896_MOESM1_ESM.pdf]

Disrupted chromatin architecture in olfactory sensory neurons by looking for the link from COVID-19 infection to anosmia

Zhen Wah Tan[1], Ping Jing Toong[1], Enrico Guarnera[1,&] and Igor N. Berezovsky[1,2,*]

[1] Bioinformatics Institute (BII), Agency for Science, Technology and Research (A*STAR), 30 Biopolis Street, Matrix, Singapore 138671, Republic of Singapore

[2] Department of Biological Sciences (DBS), National University of Singapore (NUS), 8 Medical Drive, Singapore 117597

[&]Present Address: Computational Drug Discovery, EMD Serono Research and Development Institute, Merck KGaA, 45A Middlesex Tpke, Billerica, MA, 01821, USA

[*]**Corresponding Author:**

Igor N. Berezovsky

e-mail: igorb@bii.a-star.edu.sg; phone: (65) 6478 8269; fax: (65) 6478 9047

Enhancer name	Mouse genome coordinates (mm9)			Human genome coordinates (hg38)			Near human OR gene?
	Chr	Start	End	Chr	Start	End	
**EVIA**	2	111566700	111567700	1	421646	422646	Y
**THIRA**	7	93443100	93444300	1	247647764	247648964	Y
**GAVDOS**	16	58956100	58957500	3	98333106	98334506	Y
**LIPSI**	2	36982900	36984500	9	122693320	122694920	Y
**KALYMNOS**	6	116563100	116564900	10	45049357	45051157	Y
**MILOS**	7	109661022	109662409	11	4186792	4188179	Y
**P**	7	114190700	114192100	11	6823977	6825377	Y
**IKARIA**	7	106936000	106937199	11	75035882	75037081	Y
**SYMI**	11	50812200	50813599	14	19872923	19874322	Y
**LESVOS**	14	51377700	51379500	14	20261516	20263316	Y
**SKIATHOS**	14	52957700	52959300	14	21556796	21558396	Y
**H**	14	53166900	53168700	14	21703296	21705096	Y
**CRETE**	11	73848400	73850199	17	3317487	3319286	Y
**SIFNOS**	11	87749900	87751500	17	58133191	58134791	Y
**SIKINOS**	9	18525100	18526900	19	9054150	9055950	Y
**NIMOS**	4	118314100	118315900	1	43129059	43130859	N
**KOS**	3	106678500	106680100	1	110797728	110799328	N
**FOLEGANDROS**	3	97294700	97296500	1	147359887	147361687	N
**PONTIKOS**	3	97141900	97143500	1	147494604	147496204	N
**SKORPIOS**	3	97113500	97114900	1	147527207	147528607	N
**OTHONI**	3	97111500	97113500	1	147528471	147530471	N
**CORFU**	19	14321300	14322900	9	79969332	79970932	N
**ALONISOS**	19	14314300	14315900	9	79976205	79977805	N
**AKTHRA**	19	14300700	14301900	9	79983966	79985166	N
**TINOS**	19	14170300	14171700	9	80089752	80091152	N
**RHODES**	1	94441500	94443500				
**HYDRA**	2	112049700	112051300				
**NISYROS**	3	106683500	106684900				
**SFAKTIRIA**	6	42818900	42820700				
**KEFALLONIA**	7	6497300	6499100				
**IOS**	7	115939700	115941900				
**FOURNI**	7	147372100	147374100				
**LEFKADA**	11	52014700	52015900				
**KARPATHOS**	14	50681500	50682900				
**LEMNOS**	15	98340700	98342300				

Supplementary Table S1. Mapping of Greek Islands enhancers from the mouse genome to the human genome. List of Greek Islands enhancers identified on the mm9 mouse reference genome, with genomic coordinates listed. Enhancers that have corresponding regions in the human reference genome hg38 have genomic coordinates mapped using the UCSC LiftOver tool. A mapped enhancer located near (within 1Mb from) at least one OR gene is considered as a putative enhancer in this work.

Chromosome	Sample	Energy ratio cutoff			
		**1.0**	**0.8**	**0.6**	**0.5**
**1**	c152	32.5	48.5	108.8	218.7
	c187	30.4	46.1	109.5	246.8
	p102	31.7	53.3	184.8	370.4
	p146	28.4	44.0	129.2	292.1
**11**	c152	32.9	49.4	111.7	220.8
	c187	30.8	46.3	115.9	254.5
	p102	32.5	56.4	199.4	446.1
	p146	28.4	44.2	138.0	353.7
**14**	c152	32.6	48.6	104.1	204.7
	c187	30.9	46.0	108.7	246.8
	p102	32.2	55.3	181.4	407.1
	p146	28.3	44.8	127.7	318.3

Supplementary Table S2. Average structural unit sizes at different levels of hierarchy. For each sample, we identified structural units at the whole-chromosome level with interaction energy ratios down to various cutoff values, $r_{max} = 1.0, 0.8, 0.6, 0.5$ (see also Materials and Methods for further explanations). The corresponding average size of structural units for each case is listed.

Chromosome	H	p-value
chr1	41963.10	< 1E-300
chr2	904.74	9.17E-199
chr3	51828.36	< 1E-300
chr4	47904.05	< 1E-300
chr5	3248.15	< 1E-300
chr6	13517.12	< 1E-300
chr7	18432.48	< 1E-300
chr8	757.12	1.13E-166
chr9	3209.17	< 1E-300
chr10	5779.49	< 1E-300
chr11	722.05	4.79E-159
chr12	1747.75	< 1E-300
chr13	7318.48	< 1E-300
chr14	10073.62	< 1E-300
chr15	13149.66	< 1E-300
chr16	5218.51	< 1E-300
chr17	1405.67	< 1E-300
chr18	8361.45	< 1E-300
chr19	8648.14	< 1E-300
chr20	1049.59	2.99E-230
chr21	17055.94	< 1E-300
chr22	9842.46	< 1E-300

Supplementary Table S3. Kruskal-Wallis test comparing the radial locations of each chromosome, between control and patient subjects.

Chromosomes	1-19 vs 1		1-12 vs 1		9-14 vs 9	
	D	p-value	D	p-value	D	p-value
**control187**	0.2090812	< 1E-300	0.3541915	< 1E-300	0.0349082	< 1E-300
**patient102**	0.3235534	< 1E-300	0.47059	< 1E-300	0.3517749	< 1E-300
**patient147**	0.288284	< 1E-300	0.472604	< 1E-300	0.0877885	< 1E-300

Supplementary Table S4. Kolmogorov-Smirnov test of difference between selected two-chromosome distance correlation function and the one-chromosome distance correlation function of the larger of the two chromosomes.

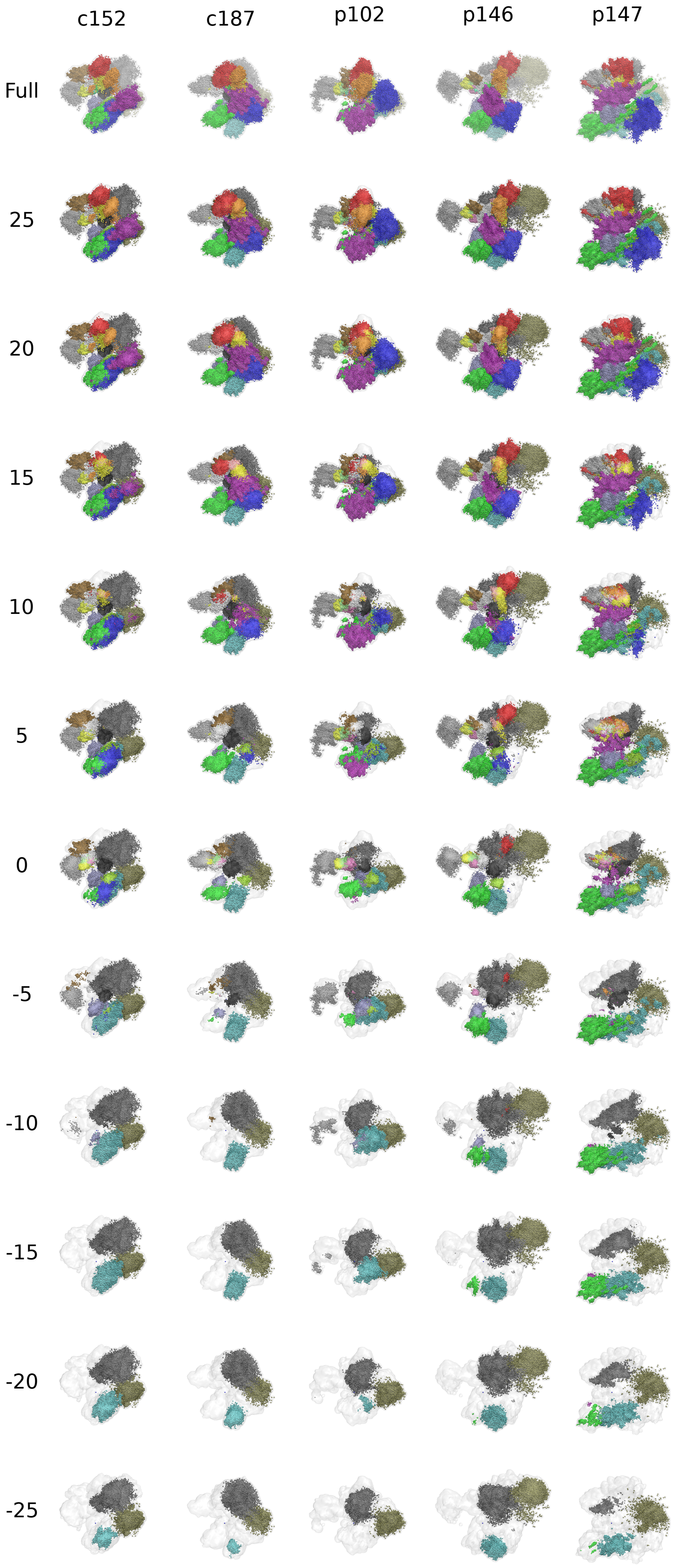
c152
c187
p102
p146
p147
Full
25
20
15
10
5
0
-5
-10
-15
-20
-25

Supplementary Figure S1. Cross-sectional views of OSN chromatin ensemble reconstructions. Cross-sectional views of chromatin ensemble reconstructions, from $z = +25$ a.u. to $z = -25$ a.u.: corresponding values of z are indicated on the left of each row.

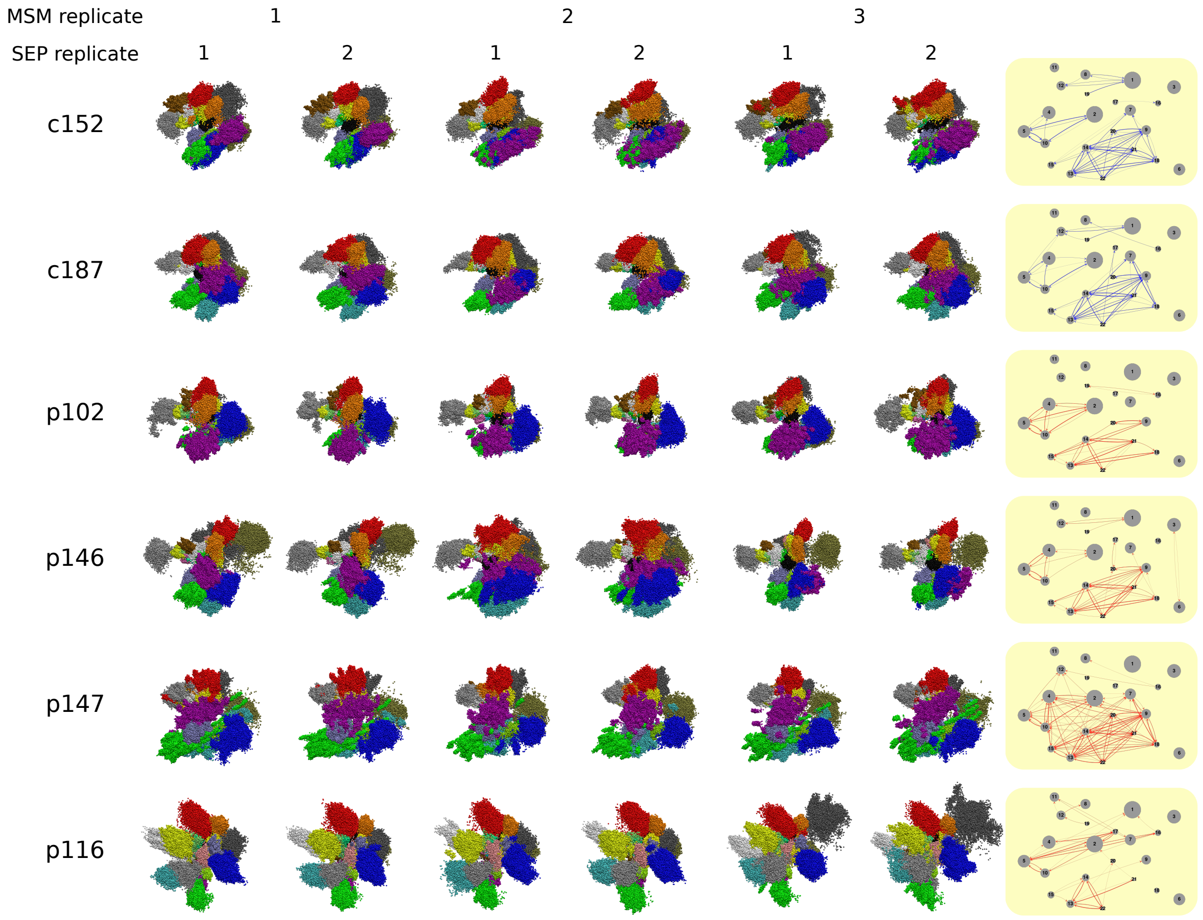

Supplementary Figure S2. Comparing MSM- and SEP-replicates of whole-genome reconstruction on patient and control OSN Hi-C data. Left. For each Hi-C sample, we performed 3 replicates of MSM-based partitioning to identify structural units, and in each case performed 2 replicates of SEP ensemble reconstructions. The ensemble structures are shown, colored by chromosomes. **Right.** Network representation of chromosomal intermingling in each Hi-C sample. Chromosome pairs with strong intermingling on average (across 6 reconstruction replicates) are joined by an edge, colored blue/red in control/patient samples.

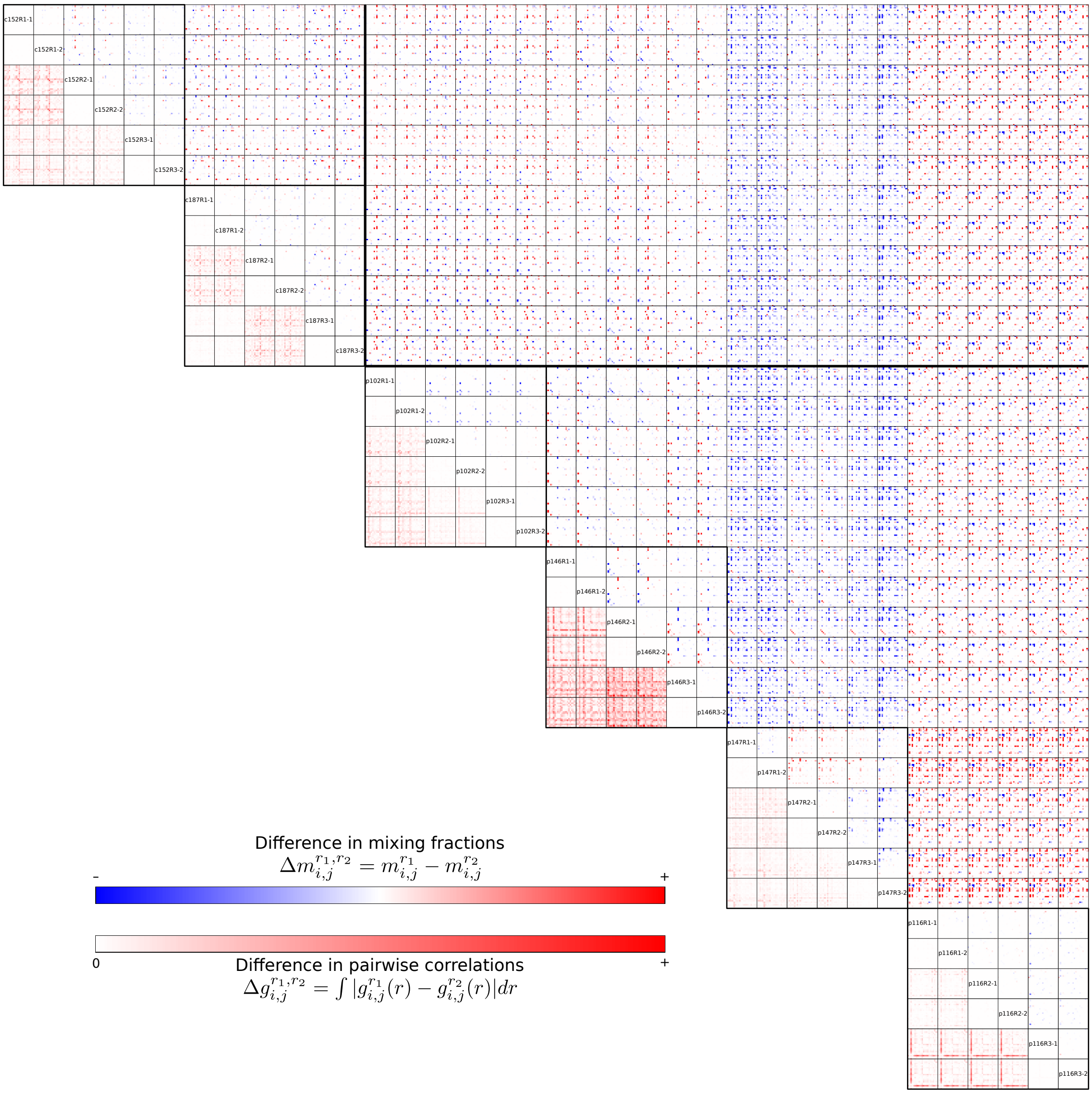

Supplementary Figure S3. Comparing structural metrics shows robustness of reconstructions. The metrics of chromatin structure between MSM and SEP replicates of the same Hi-C sample, and between Hi-C samples were compared. On the lower left side of the diagonal, comparison of reconstruction replicates on the same Hi-C sample by plotting the heatmap of histogram differences in pairwise correlation functions $\Delta g_{ij}^{r_1,r_2}$ is presented. On the upper right side of the diagonal, the differences in mixing fractions $\Delta m_{ij}^{r_1,r_2}$ are plotted.

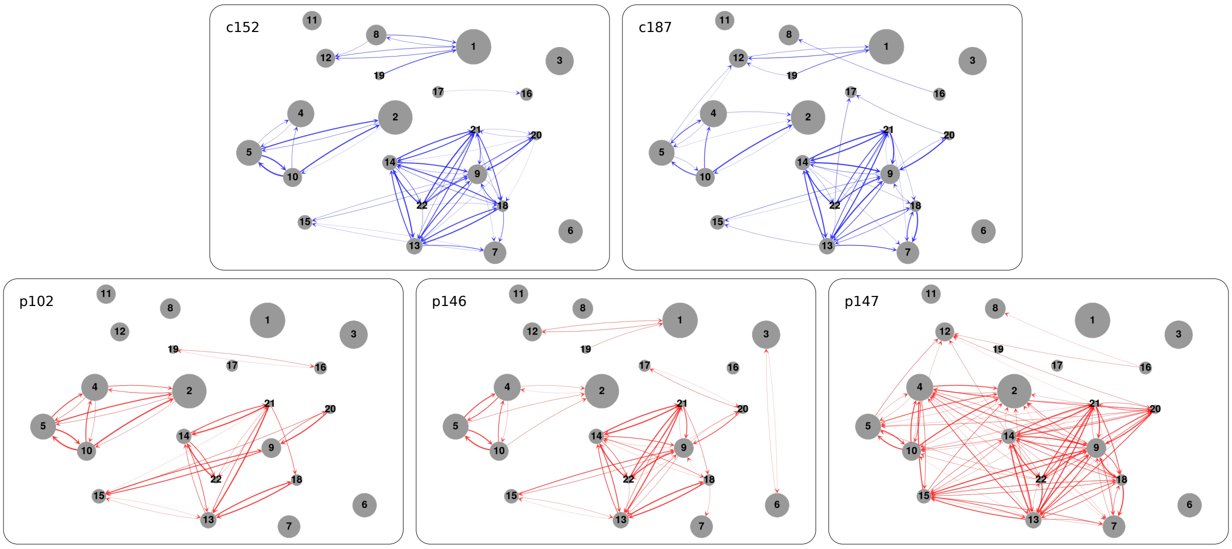

Supplementary Figure S4. Chromosomal intermingling network for all Hi-C samples. Network of strongly intermingling chromosomal territories in each sample. For each sample, edges connecting CTs $i \rightarrow j$ indicate that more than 10% of chromosome i is located within 1 a.u. from chromosome j.

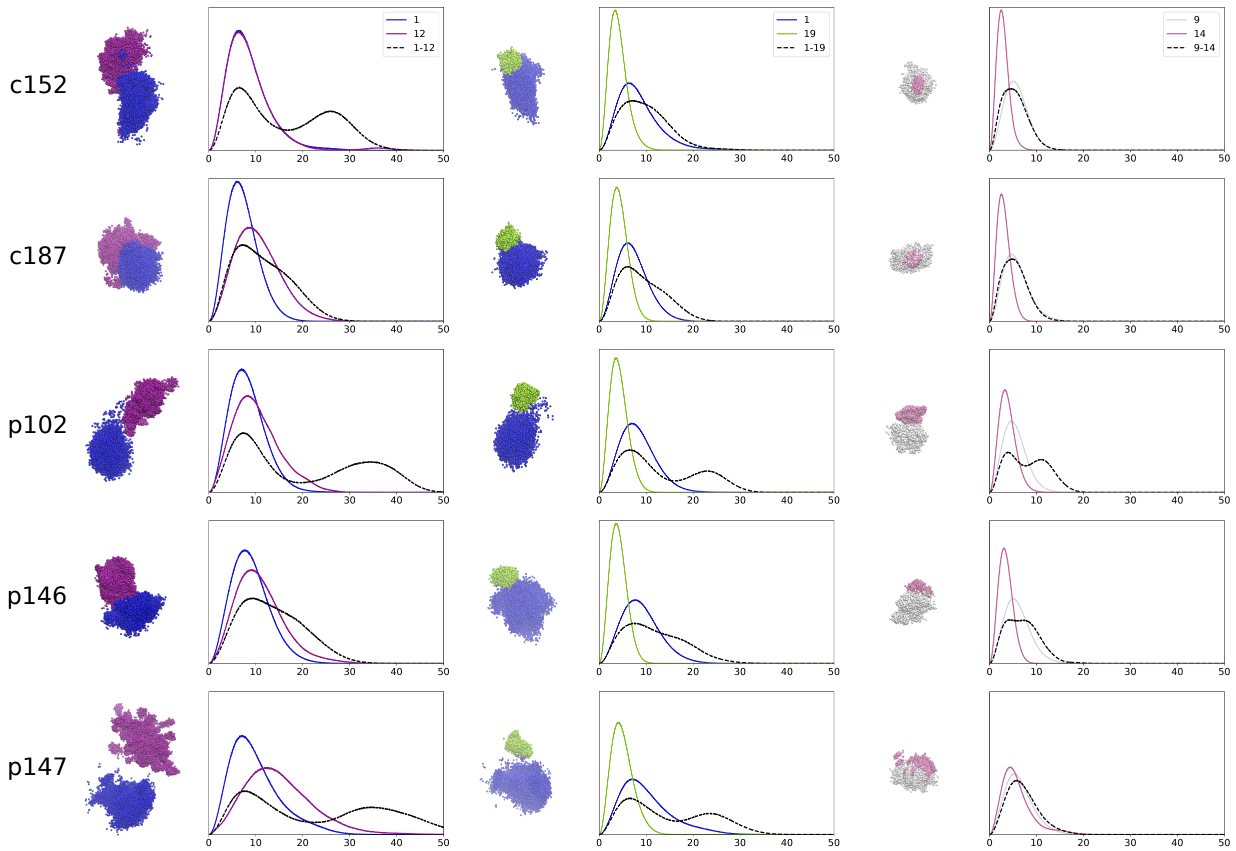

Supplementary Figure S5. Chromosomal territory morphology and distance correlation plots for sample CT pairs for all Hi-C samples). Structures and $g(r)$ plots are shown here for all samples.

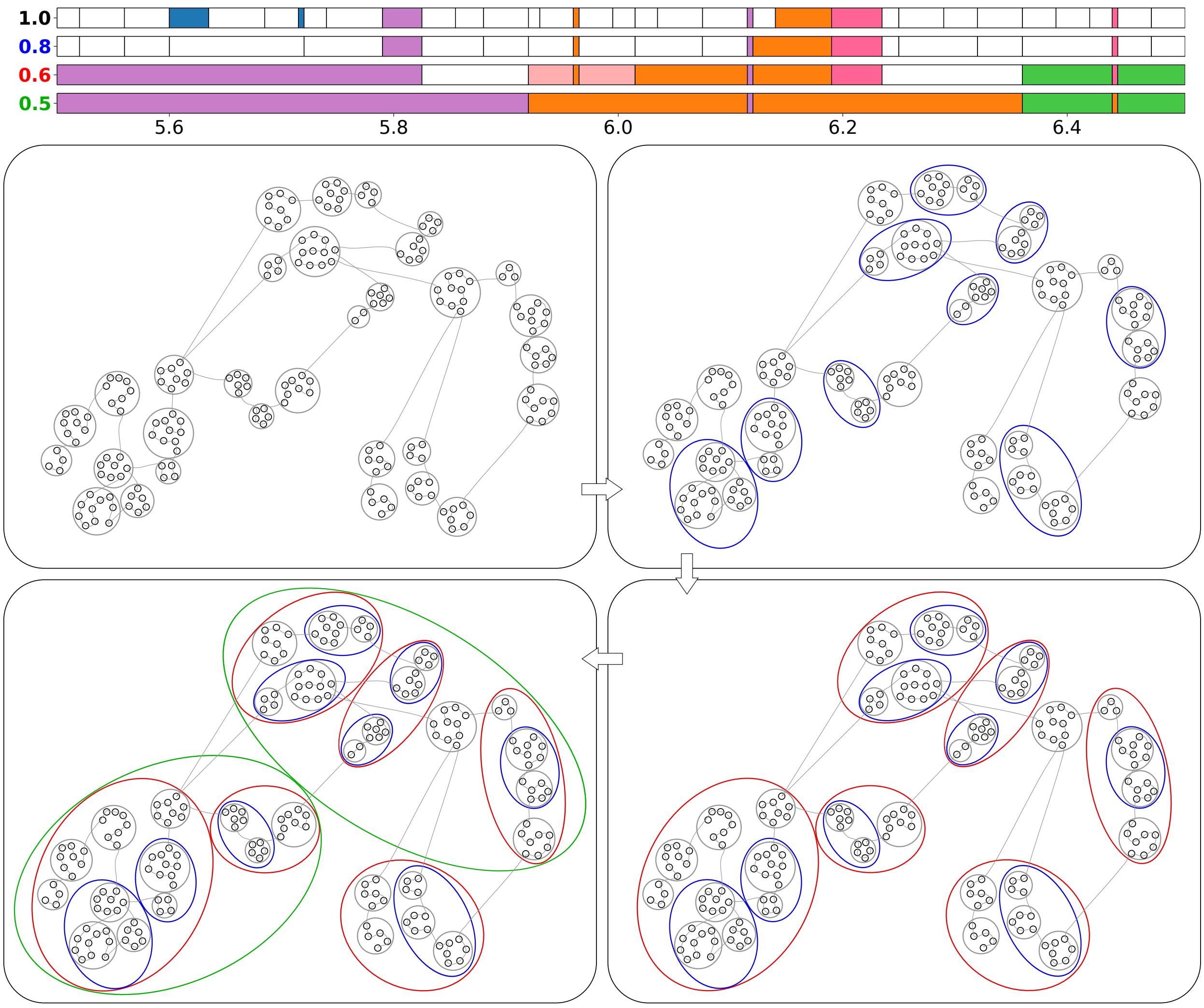

Supplementary Figure S6. Iterative procedure for identifying local hierarchy of the fine structural units. This Supplementary File, Figure Shows Structural units identified at various levels of hierarchy in a 1Mb region of chromosome 11 (5.5-6.5Mb) are shown here. Individual 5kb bins/loci in this region are represented by black circular nodes, with a chromosomal trace overlaid to show the relative genomic locations of each locus. Beginning by considering each locus as an individual structural unit, we iteratively join pairs of structural units with the highest interacting energy ratios r, to a point where the largest energy ratio r_{max} falls below 1.0, yielding structural units denoted by the grey ellipses, in the first panel on the top left. Continuing with this procedure, we obtain a deeper level of hierarchy when r_{max} falls below 0.8, where some of the grey units are merged into larger units marked by the blue ellipses in the second panel to the right. This iterative procedure can be applied further to obtain better-separated structural units with lower values of $r_{max} = 0.6, 0.5$, merging to form larger units marked by red and green ellipses respectively. The 1D representations of the structural units (shown in the top) obtained at each level of hierarchy are denoted by the value of r_{max} on the left. Units composed of a single contiguous genomic segment are shown as white horizontal bars in the plot, while units composed of non-contiguous segments are color-coded accordingly: *e.g.,* the two blue segments and the two orange segments at $r_{max} = 1.0$ form two corresponding structural units.

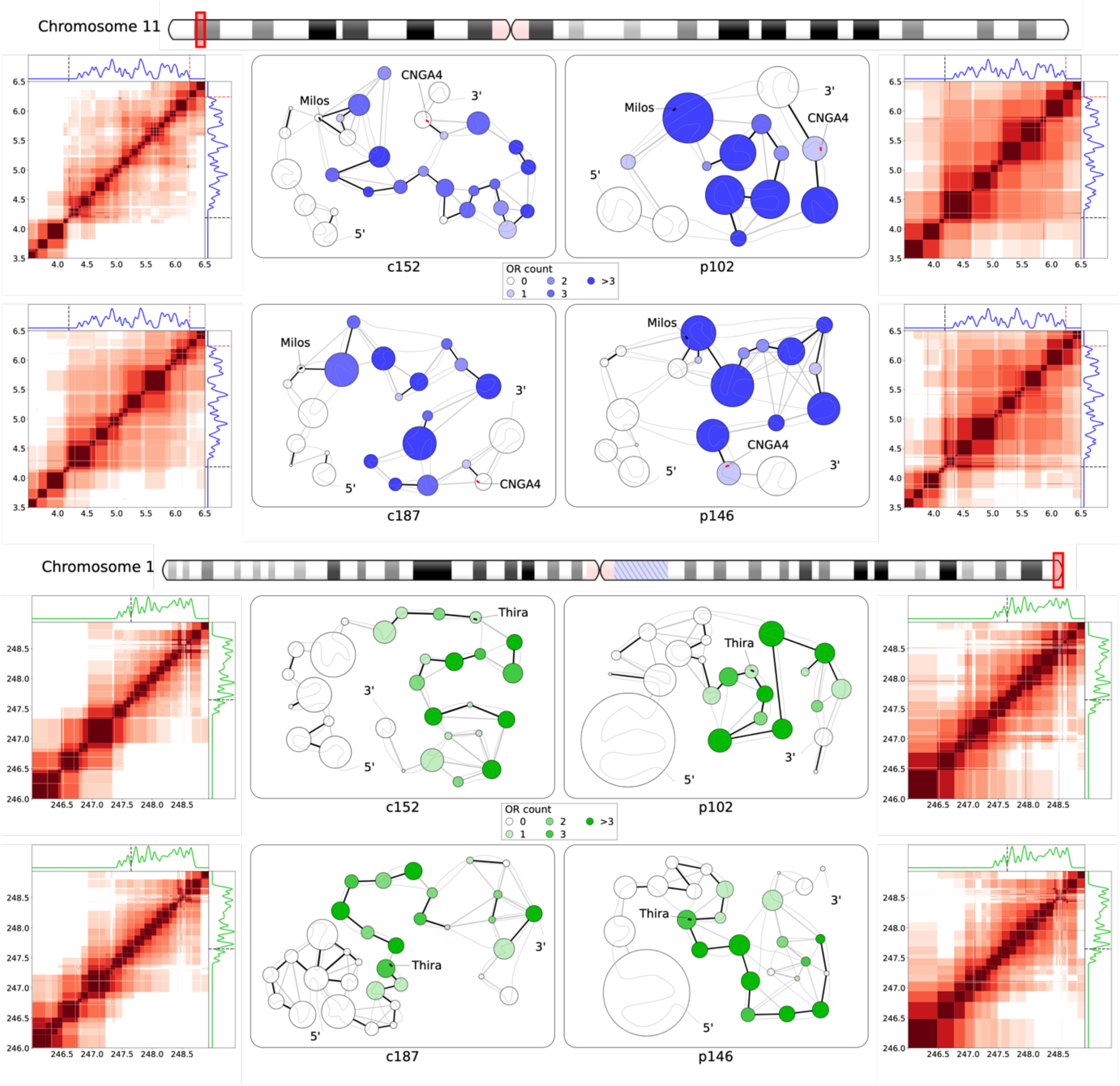

Supplementary Figure S7. Comparison of fine structural organizations in the Class I and II olfactory receptor gene clusters on chromosome 11 and 1, respectively. This figure is a compillation of Figrues 4 and 5, helping to compare structural organizations of chromosome regions containing Class I and II OR genes.
